# Propofol induces proliferation and invasion of gallbladder cancer cells through activation of Nrf2

**DOI:** 10.1186/1756-9966-31-66

**Published:** 2012-08-19

**Authors:** Lingmin Zhang, Ning Wang, Suna Zhou, Wenguang Ye, Guixia Jing, Mingxin Zhang

**Affiliations:** 1 Department of Anesthesiology, First Affiliated Hospital, Medical School, Xi'an Jiaotong University, Xi’an, Shaanxi Province, 710061, China; 2 Department of Anesthesiology, Second Affiliated Hospital, Medical School, Xi'an Jiaotong University, Xi’an, Shaanxi Province, 710061, China; 3 Department of Thoracic Oncosurgery, First Affiliated Hospital, Medical School, Xi'an Jiaotong University, Xi’an, Shaanxi Province, 710061, China; 4 Department of Gastroenterology, Tangdu Hospital, Fourth Military Medical University, Xi'an, Shaanxi Province, 710038, China

**Keywords:** Propofol, NF-E2-related factor 2 (Nrf2), Gallbladder cancer, Proliferation, Invasion

## Abstract

**Background:**

Propofol is one of the most commonly used intravenous anaesthetic agents during cancer resection surgery, but the effect of propofol on gallbladder cancer is not clear. NF-E2-related factor 2 (Nrf2) is abundantly expressed in cancer cells and relates to proliferation, invasion, and chemoresistance. The aims of the current study were to evaluate effects of propofol on the behavior of human GC cells and role of Nrf2 in these effects.

**Method:**

The effects of propofol on cell proliferation, apoptosis, and invasion were detected by MTT assays, flow cytometry, and transwell assay. Also, activation of Nrf2 was determined by western blot, RT-PCR, and immunofluorescence assays. Nrf2 was knocked-down in GBC-SD cells by shRNA before evaluating the role of Nrf2 in the influence of propofol on biological behaviors.

**Results:**

Propofol promoted the proliferation of GBC-SD cells in a dose- and time- dependent manner. After exposure to propofol for 48 h, GBC-SD cells showed decreased apoptosis and increased invasion. Also, propofol over-expressed Nrf2 at both the protein and mRNA levels and induced translocation of Nrf2 into the nucleus. Finally, loss of Nrf2 by shRNA reversed the effect of propofol on cell proliferation, apoptosis, and invasion.

**Conclusion:**

Propofol induces proliferation and promotes invasion of GC cells through activation of Nrf2.

## Introduction

Propofol (2,6-diisopropylphenol), one of the most commonly used intravenous anesthetic agents producing smooth induction and rapid recovery from anesthesia, has gained wide acceptance since its introduction in the late 80s [1]. Apart from its multiple anesthetic advantages, propofol exerts a number of non-anesthetic effects [2]. Interestingly, propofol has antioxidant effects and preserves the endogenous organ protective against ischemic or hypoxic injury. Heme oxygenase-1 (HO-1) is involved in the mechanisms for organ protection function of propofol [3-6]. However, HO-1 plays an important role in cancer [7,8]. Some studies have suggested a possible correlation between propofol and cancers, but the results are undefined [9-14]. Some studies revealed that clinically relevant concentrations of propofol increased the migration of breast carcinoma cells by activation of GABA [9]. On the other hand, opposite results suggested that these concentrations of propofol inhibited the invasion of human cancer cells by modulating Rho A or ERK1/2 [10,11]. Other studies have demonstrated the effect of propofol on immune response and metastasis in *in vivo* experiments [12-14]. Considering the widely use of propofol in clinic setting, it would be of great importance to investigate the relationship between propofol and cancer.

NF-E2-related factor 2 (Nrf2) is a key transcription regulator for antioxidant and detoxification enzymes, of which HO-1 is the most important one [15,16]. Recent studies find that Nrf2 is abundantly expressed in cancer cells and relates to proliferation, invasion, and chemoresistance [17]. Our early observations also found that expression of Nrf2 was up-regulated in gallbladder cancer (GC) tissues and served as an independent prognostic factor [18].

Propofol has antioxidant properties partly through up-regulation of HO-1, a downstream target gene of Nrf2. We tested the hypothesis that propofol activates Nrf2, hence it affects the progression of cancer. The aims of the current study were to evaluate effects of propofol on the behavior of human GC cells and role of Nrf2 in these effects.

## Materials and methods

### Cell culture and reagents

Gallbladder carcinoma cells (GBC-SD) were obtained from Shanghai Institute of Cell Biology, Chinese Academy of Sciences. Cells were cultured in RPMI 1640 media (Sigma, St. Louis, USA), supplemented with 10% fetal bovine serum and 100 units/mL of penicillin and streptomycin at 37°C in a humidified 5% CO_2_. Propofol was purchased from Aldrich (Milwaukee, WI). Propofol was diluted in dimethyl sulfoxide (DMSO, Sigma, St. Louis, MO, USA) for *in vitro* assays.

### Cell growth assay

The cells were seeded at a density of 5 × 10^3^ cells/well in 96-well plates at a final volume of 180 μL in incubation, at 37°C, with 5% CO2. After different time incubation, 20 μL of 5 mg/mL solution of MTT (Sigma, St. Louis, MO, USA) in 1× PBS was added to each well. The plates were then incubated for 4 h at 37°C. The reaction was then solubilized in 100% DMSO, 20 μl/ well, and shaken for 15 min. Absorbance of each well was measured on a multidetection microplate reader (BMG LABTECH, Durham, NC, USA) at a wavelength of 570 nm.

### Apoptosis analysis

The cells were washed twice with cold 10 mM 1× PBS and resuspended in 1× binding buffer (BD Biosciences, San Jose, CA, USA). Apoptosis in GC cells was quantified by staining with annexin V-fluorescein isothiocyanate (FITC) and propidium iodide (PI) [annexin V-Phycoerythrin (PE) and 7-amino-actinomycin D (7-AAD) for apoptosis analysis for cells transfected by ShRNA vectors with the GFP fluorescence] The samples were analyzed using flow cytometry (FACSCalibur, BD Biosciences, San Jose, CA).

### Cell invasion assay

For invasion assay, the membrane invasion culture system (transwell membranes of 6.5 mm diameter and 8 μm pore size; Costar) was used according to the standard protocol. Briefly, harvested cells (1 × 10^5^) resuspended in 100 μL of serum free RPMI 1640 were added into the upper compartment of the chamber. A total of 1000 μL conditioned RPMI 1640 medium with 20% (v/v) fetal bovine serum was used as a source of chemoattractant and placed in bottom compartment of chamber. After 48 hours, the noninvasive cells on the upper surface of the membrane were removed with a cotton swab. The transformed cells that migrated through the Matrigel matrix and stuck to the lower surface of the membrane were fixed with 4% paraformaldehyde, stained with 1% crystal purple. The invasive cells were then counted (five high-power fields/chamber) using an inverted microscope (Olympus, Lake Success, NY). Each tests repeated in triplicate.

### RNA extraction and quantitative reverse transcription-PCR (qRT-PCR)

Total RNA was isolated with Trizol reagent (Invitrogen, Carlsbad, CA, USA) following the manufacturer’s instruction. qRT-PCR was carried out using a BioRad iQ5 Real-Time PCR Detection System to confirm the expression levels of mRNAs. In brief, the reverse transcription reaction was carried out in a 20 μl volume with 1 μg of total RNA, by incaution at 16°C for 30 min, 42°C for 42 min, and 85°C for 5 min. 1 μl of the RT product was used in each PCR. The PCR cycling began with template denature at 95°C for 5 min, followed by 40 cycles of 95°C for 10 sec, 60°C for 20 sec, 72°C for 20 sec, and 78°C for 20 sec. Final PCR products were resolved in agarose gen electrophoresis and a single band of expected size indicated the specificity of the reaction. Relative quantification was performed using the 2^-ΔΔCT^[19]. Each PCR amplification was performed in triplicate to verify the results. The Nrf2 primers were as follows: upstream 5^′^-ACACGGTCCACAGCTCATC-3^′^; and downstream 5^′^-TGCCTCCAAGTATGTCAATA-3^′^. The GAPDH primers were as follows: upstream 5^′^-ACCACAGTCCATGCCATCAC-3^′^; and downstream 5^′^-TCCACCACC CTGTTGCTGTA-3^′^.

### Western blot analysis

Anti-Nrf2, anti-HO-1 and anti-β-actin antibodies were obtained from Santa Cruz Biotech (Santa Cruz, CA, USA). For Western blot analyses, 20 μg of total protein were electrophoresed on a 10% SDS-PAGE gel, transferred onto to PVDF membrane, blocked, and then incubated with primary antibody as indicated above. Corresponding horseradish peroxidase (HRP)-conjugated secondary antibody was then used on them at room temperature for 2 h. After chemiluminescence reaction with enhanced ECL detection reagents (Amersham, Little Chalfont, Buckinghamshire, England) according to the manufacturer’s instructions, the membranes were visualized by exposure to X-ray film in dark. Densitometric analysis was performed using Scion Image software (Scion Corporation, Frederick, MD).

### Immunofluorescence assay

GBC-SD cells (5 × 10^4^ cells/mL) were grown on coverslips in 24-well plates, with or without propofol stimulation. The cells were washed with cold PBS, fixed in 4% paraformaldehyde, permeabilized with 0.3% Triton X-100, and blocked with 5% bovine serum albumin (BSA), followed by detection of Nrf2. After incubation with primary antibodies against Nrf2 at 4°C overnight, cells were labeled using FITC-conjugated secondary antibody (Santa Cruz Biotechnology, Santa Cruz, CA). Finally, cells were stained with DAPI (1 μg/ml, Roche, Shanghai, China) for nuclear visualization. Immunoreactivity of each sample was observed using a fluorescence microscope (Olympus, Tokyo, Japan).

### shRNA design and transfection

The sequences of the shRNAs were as follows: shRNA 867: 5^′^-CACCGGTTGCCCA CATTCCCAAATCTTCAAGAGAGATTTGGGAATGTGGGCAACCTTTTTTG-3^′^; shRNA 1118: 5^′^-CACCGGGAGGAGCTATTATCCATTCTTCAAGAGAGAATGGA TAATAGCTCCTCCCTTTTTTG-3^′^; shRNA 1757: 5^′^-CACCGGGATATGGTACAA CCCTTGTTTCAAGAGAACAAGGGTTGTACCATATCCCTTTTTTG-3^′^; shRNA 2019: 5^′^-CACCGCAGTTCAATGAAGCTCAACTTTCAAGAGAAGTTGAGCTTC ATTGAACTGCTTTTTTG-3^′^. The pGP U6-shRNA plasmids were constructed by cloning the respective shRNAs into the pGPU6/GFP/ Neo vector (GenePharma, Shanghai, China). An unrelated shRNA sequence (5^′^-CACCGTTCTCCGAACGTGT CACGTCAAGAGATTACGTGACACGTTCGGAGAATTTTTTG-3^′^), with no homology to any human gene, was used as a negative control (shNC).

GBC-SD cells were seeded in a 24-well plate at a concentration of 1 × 10^5^ cells per well. Lipofectamine 2000 (Invitrogen, Carlsbad, CA, USA) was used for transfection according to the instructions. Fresh growth medium was changed 6 h after transfection and 48 h after transfection the cells were harvested for analysis. The shNC was used as a negative control. To verify the knockdown efficiency, mRNA and protein of transfected cells were collected for qRT-PCR and western blot analysis as described above. Verification of Nrf2 knockdown was determined by normalizing the levels of Nrf2 to the control.

### Statistical analysis

Data are expressed as the mean ± standard error from at least 3 separate experiments performed in triplicate. Differences between groups were assessed by unpaired, two-tailed Student’s *t* test, P < 0.05 was considered significant.

## Results

### Effect of propofol on cell proliferation, apoptosis, and invasion

**Figure 1:**
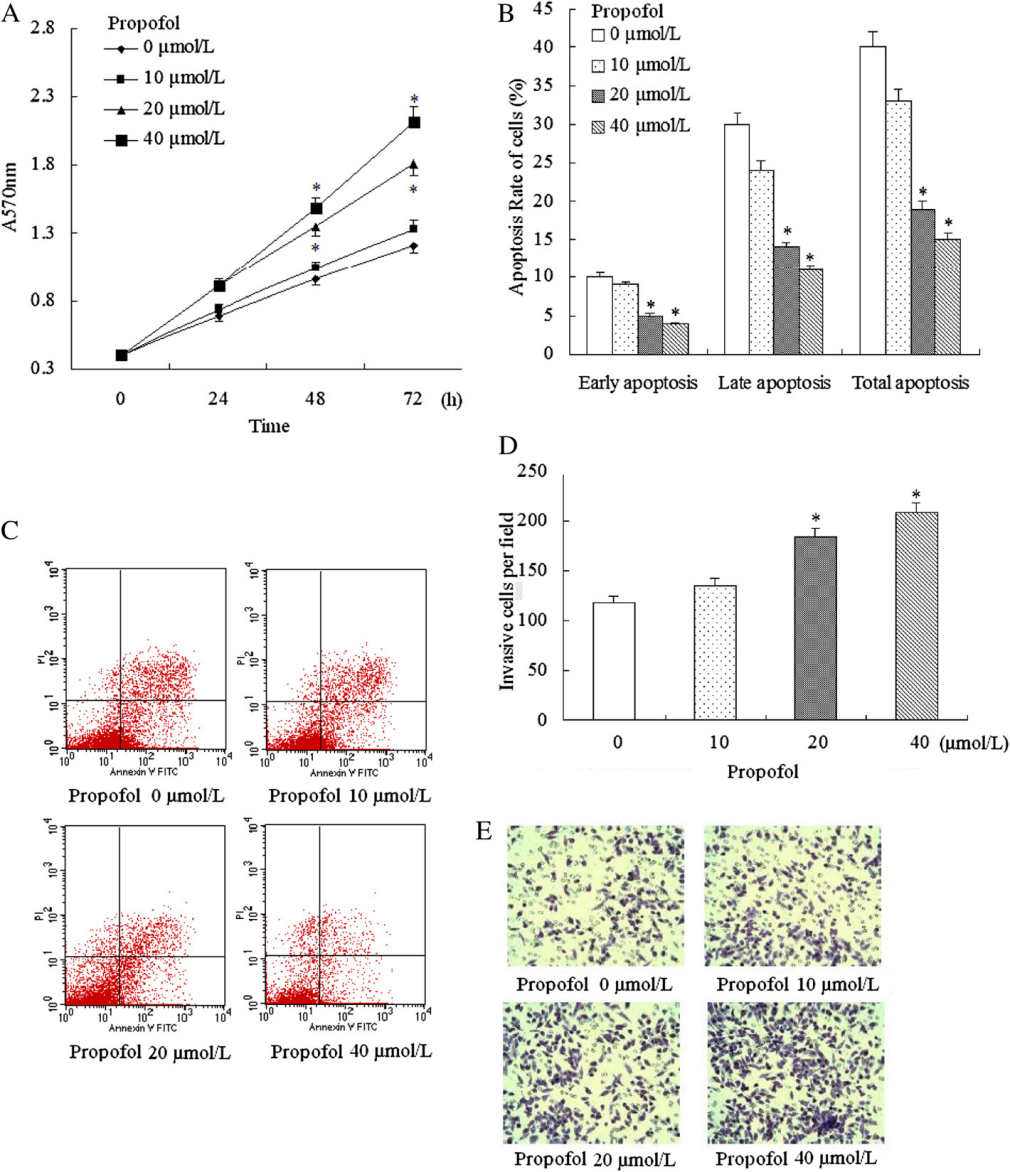
Effects of propofol stimulation on cell proliferation, apoptosis, and invasion.

We first investigated the effects of propofol on cell proliferation, apoptosis, and invasion. The GBC-SD cell lines were cultured in the presence of various concentrations of propofol and the cell proliferation were measured by the MTT assays. As shown in Figure 1A, the proliferation of GBC-SD were promoted by propofol in dose- and time- dependent manners. Propofol with the concentration 20 μmol/L and 40 μmol/L significantly promoted the proliferation at 48 h and 72 h. To further quantify the cell death, annexin V/PI analysis was performed. After exposed to propofol for 48 h, GBC-SD cells showed decreasing apoptosis (Figure 1B and Figure 1C). Cell invasion assay also revealed that propofol significantly stimulated invasion when giving a concentration of 20 μmol/L and 40 μmol/L (Figure 1D and Figure 1E). So, we chose propofol with the concentration 20 μmol/L in the following experiments.

Cells were incubated with increasing concentrations of propofol (0–40 μmol/L). (A) Propofol increased GBC-SD cells proliferation in a time- and dose-dependent manner. (B) and (C) Apoptosis analysis using flow cytometry showed that propofol inhibited the apoptosis. (D) and (E) Cell invasion assay revealed that propofol significantly stimulated invasion. All of these results confirmed that propofol (given a concentration greater than or equal 20 μmol/L) significantly promoted proliferation, inhibited apoptosis, and stimulated invasion. * P < 0.05 vs. Control (cells without propofol exposure).

### Activation of Nrf2 by propofol stimulation

**Figure 2:**
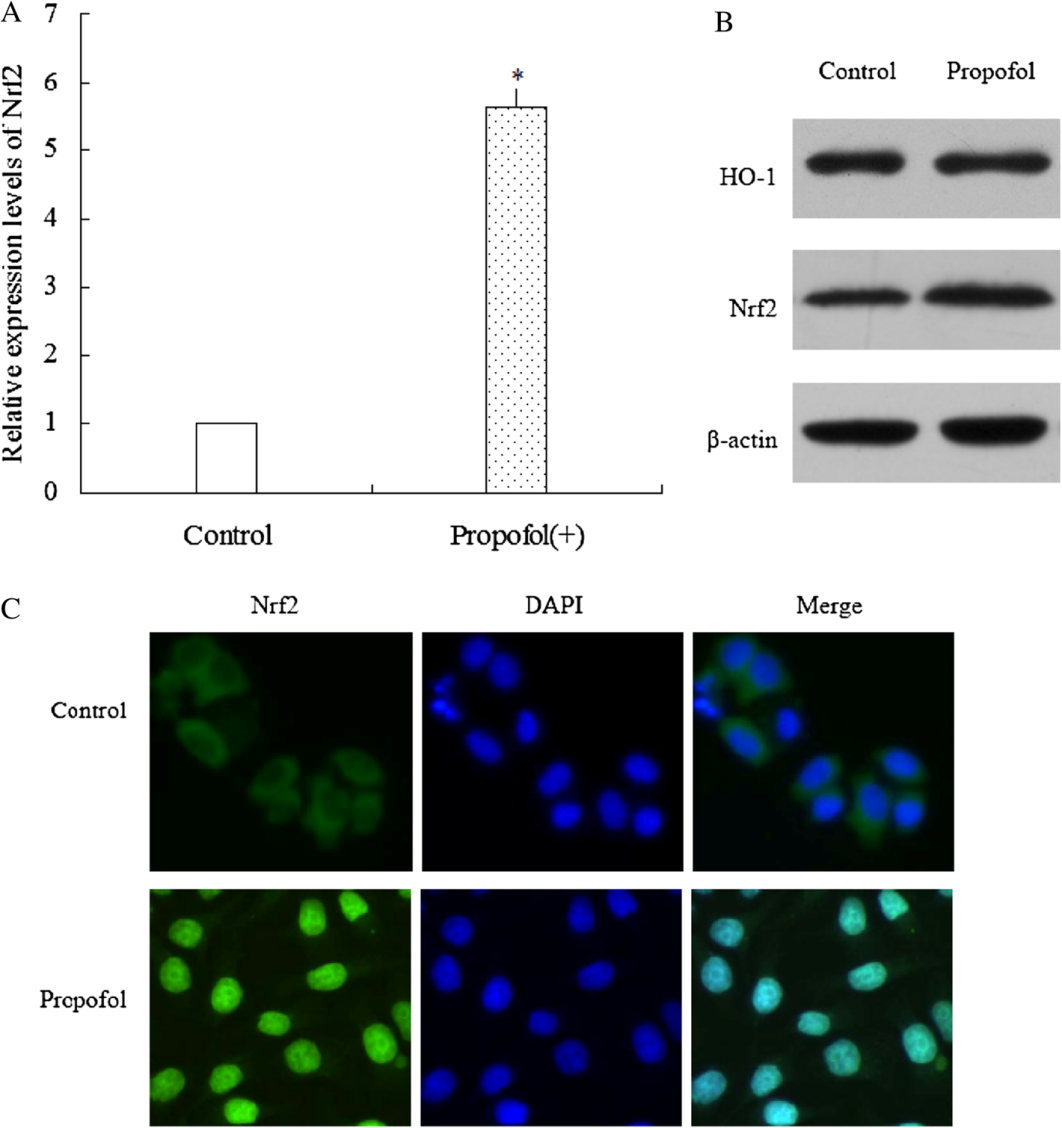
Activation of Nrf2 by propofol stimulation.

We then evaluated the effect of propofol stimulation on activation of Nrf2 in mRNA and protein levels. The results showed that exposing to propofol (20 μmol/L) for 48 h up-regulated the expression of Nrf2 at mRNAs levels (Figure 2A). Besides, exposing to propofol (20 μmol/L) for 48 h also up-regulated the protein expression of both HO-1 and Nrf2 (Figure 2B). Moreover, cells exposed to propofol showed translocation of Nrf2 into the nucleus (Figure 2C).

(A) After stimulating by propofol, Nrf2 mRNA levels were quantified by real-time PCR analysis. Data were normalized by using GAPDH as an internal standard. * P < 0.05 vs. Control (cells without propofol exposure). These experiments were performed in triplicate. (B) After stimulating by propofol, HO-1 and Nrf2 protein level was analyzed by western blot. β-actin expression was monitored as the internal standard. (C) After stimulating by propofol, subcellular location of Nrf2 was detected by immunofluorescence assay. Propofol stimulation increased translocation of Nrf2 into the nucleus.

### Knock-down of Nrf2 by specific shRNAs

**Figure 3:**
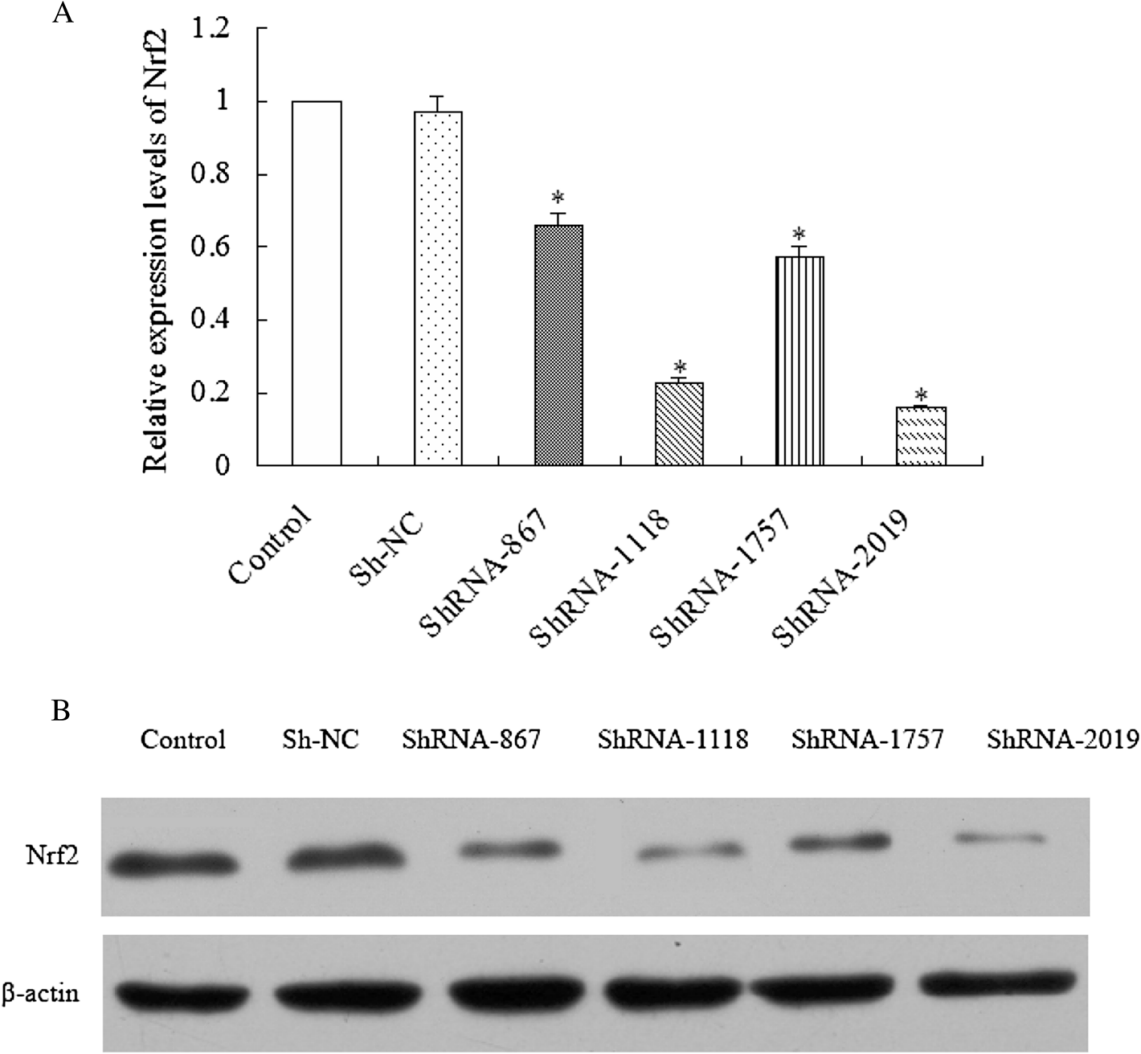
Knock-down of Nrf2 by specific shRNAs.

In order to knock down Nrf2, we constructed Nrf2-shRNA recombinant plasmids and transfected them into GBC-SD cells to knockdown the expression of Nrf2. qRT-PCR and western blot showed that Nrf2 expression was dramatically down-regulated at both the mRNA and protein levels in GBC-SD cells compared with parental cells and Sh-NC (Figure 3A and Figure 3B). Among the four recombinant plasmids, ShRNA-1118 and ShRNA-2019 has the highest suppression efficiency, so both of them were used to process the following experiments.

Forty-eight hours after transfection, cells were harvested. (A) Nrf2 mRNA levels were quantified by real-time PCR analysis. Data were normalized by using GAPDH as an internal standard. *P < 0.05 vs. Control (parental cells). (B) Nrf2 protein level was analyzed by western blot. β-actin expression was monitored as the internal standard.

### Loss of Nrf2 reverses the effects of propofol on cell proliferation, apoptosis, and invasion

**Figure 4:**
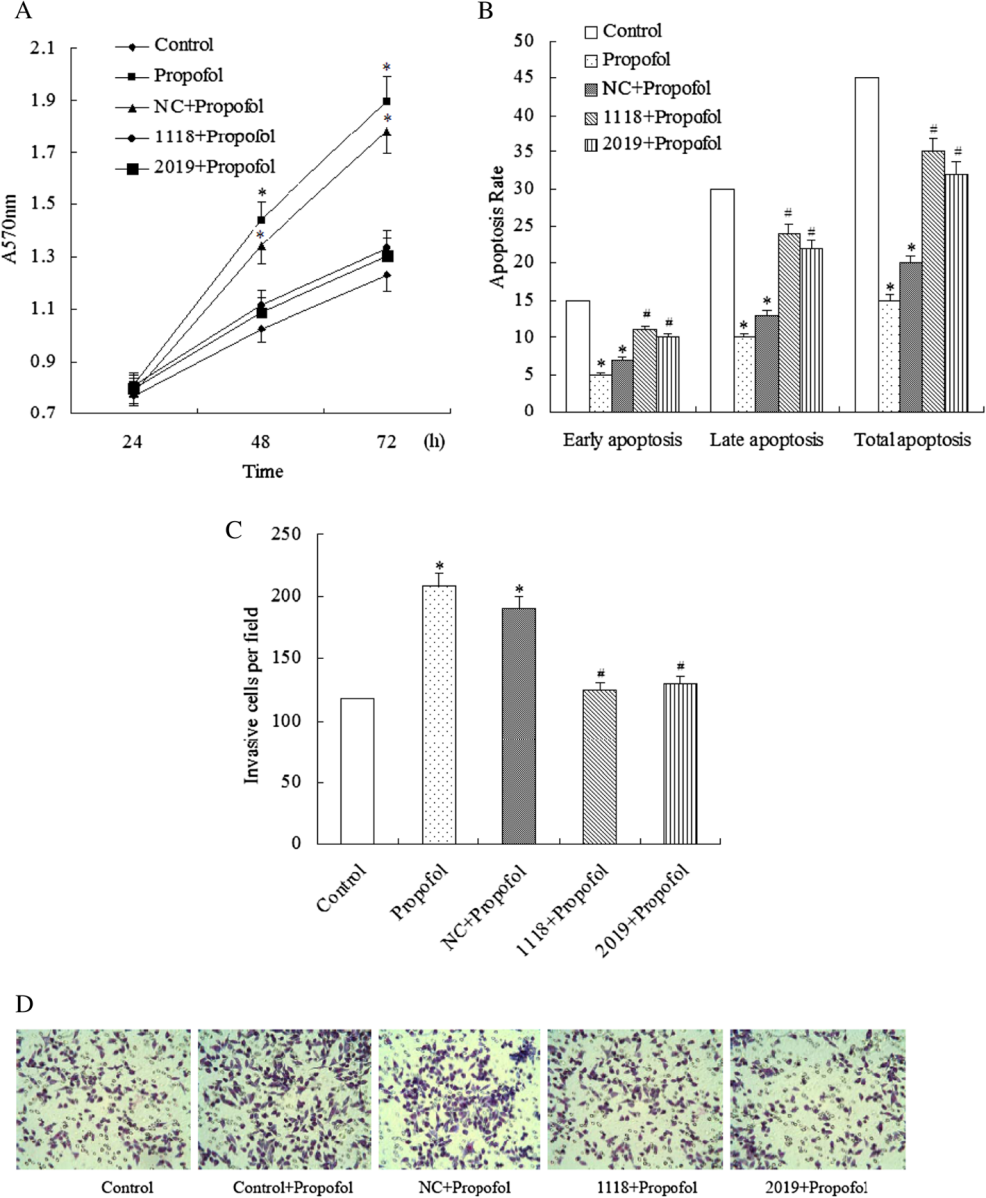
Regulation of loss of Nrf2 for the effects of propofol on cell proliferation, apoptosis, and invasion.

Finally, we examined whether loss of Nrf2 reversed the effects of propofol on cell proliferation, apoptosis, and invasion. Results showed that propofol alone and propofol plus sh-NC significantly promoted proliferation, stimulated invasion and inhibited apoptosis compared to parent cells. In contrast, propofol with ShRNA-1118 and ShRNA-2019 reversed these effects (Figure 4A to Figure 4D).

After transfected by different vectors, GBC-SD cells were incubated with propofol (20 μmol/L). (A) and (B) The results of cell proliferation assay showed propofol alone and propofol puls with sh-NC significantly promoted proliferation and inhibited apoptosis, while propofol with ShRNA-1118 and ShRNA-2019 reversed these effects. (C) and (D) Cell invasion assay demonstrated that loss of Nrf2 reversed the effect of propofol on invasion: propofol alone and propofol plus sh-NC significantly stimulated invasion, while propofol with ShRNA-1118 and ShRNA-2019 suppressed invasion in GBC-SD cells. Each experiment was performed three times in triplicate. * P < 0.05 vs. Control, # P < 0.05 vs. Propofol. Control: parental cells; Propofol: parental cells with propofol; NC + Propofol: cells transfected by ShNC incubated with propofol; 1118 + Propofol: cells transfected by ShRNA-1118 incubated with propofol; 2019 + Propofol: cells transfected by ShRNA-2019 incubated with propofol.

## Discussion

We evaluated effects of propofol on the behavior of human GC cells and the role of Nrf2 in these effects. Our study showed that propofol induced proliferation and invasion of gallbladder cancer cells through activation of Nrf2.

Anesthesia represents one of the most important medical advances in history and is widely considered safe. Nevertheless, numerous anesthetics are used for cancer resection even if their effect on the behavior of cancer cells is unclear [20]. Propofol is one of these anesthetics. In *in vivo* experiments, different kinds of cancer cells treated by different concentrations of propofol showed divergent results. Garib et al. found that propofol (34 μmol/L) increased migration of MDA-MB-468 breast carcinoma cells [9]. In contrast, Mammoto et al. demonstrated that clinically relevant concentrations of propofol (5.6-28 μmol/L) decreased the invasion ability of human cancer cells (HeLa, HT1080, HOS and RPMI-7951) [10]. Also, Miao et al. reported that propofol (at 45 μmol/L) stimulation inhibited invasion of LOVO colon cancer cells [11]. So we set a concentration range of propofol (0–40 μmol/L) to test its effect on the behavior of GBC-SD cells. Our results showed that propofol stimulation promoted proliferation by inhibiting apoptosis and increased the invasion ability.

Nrf2 belongs to the cnc (“cap ‘n’ collar”) subfamily of the basic region leucine zipper transcription factors [21]. Nrf2 is a critical factor regulating cellular defense response in many human pathological conditions. Upon exposure of cells to oxidative stress or chemopreventive compounds, Nrf2 translocates to the nucleus to activate transcription of several different types of genes, including those encoding endogenous antioxidants, phase II detoxifying enzymes, and transporters [22]. As one of Nrf2 downstream target genes, HO-1 is an antioxidant enzyme that degrades prooxidant heme into ferrous iron, carbon monoxide, and biliverdin [16]. HO-1 participates in the mechanisms for organ protection function effect of many intravenous and inhaled anesthetics including propofol [5]. Since HO-1 is up-regulated by Nrf2 and propofol, we then investigated whether propofol had an effect on the activation of Nrf2. We found that propofol increased the expression of Nrf2 both in mRNA and protein levels. Further, immunofluorescence assay also confirmed that Nrf2 translocated to nucleus after exposed to propofol.

Recent data has revealed the other side of Nrf2. Nrf2 over-expressed in many types of human cancer, giving cancer cells an advantage for survival and growth. Further studies show various genetic abnormalities of the Nrf2 repressor, Keap1, in several cancer cell lines and tumor tissues, including GC. Our previous studies also demonstrated that Nrf2 was up-regulated in GC tissues and high expression of Nrf2 related to poorer survival [18]. Thus, we next evaluated the role of activation of Nrf2 by propofol in its effect on behavior of human GC cells. Through knockdown of expression of Nrf2 by shRNA, the effect of propofol on proliferation and apoptosis were reversed.

One important limitation of our study is short of *in vivo* studies. There are also confused results about effect of propofol on immune response and metastasis *in vivo* experiments [12-14]. It would be interesting and important to clear the exact effect of propofol on GC in animal model and clinic. These will be further explored in future studies.

In conclusion, this study provides new insights into effect of propofol on behavior of GC cells and the related mechanism. Our present study suggests that propofol induces proliferation and promotes invasion of GC cells through, at least partly, activation of Nrf2. It might therefore be speculated that propofol might not be the appropriate anaesthetic drug in the surgery of GC patients. However, this should be verified in further studies, including animal trials and prospective clinical studies.

## Competing interests

No authors of this manuscript have any competing interests to disclose.

## Authors’ contributions

LM and NW participated in the design and conduction of experiments, data analysis, and final drafting and writing of the manuscript. LM, NW, SZ and WY all contributed for these experiments. GJ and MZ was closely involved in research design and drafting of the final manuscript. All authors read and approved the final manuscript.
